# The role of affect and reward in the conflict-triggered adjustment of cognitive control

**DOI:** 10.3389/fnhum.2012.00342

**Published:** 2012-12-31

**Authors:** Gesine Dreisbach, Rico Fischer

**Affiliations:** ^1^Department of Psychology, University of RegensburgRegensburg, Germany; ^2^Department of Psychology, Technical University DresdenDresden, Germany

**Keywords:** cognitive control, conflict adaptation, affect, reward, conflict monitoring

## Abstract

Adapting to changing task demands is one of the hallmarks of human cognition. According to an influential theory, the conflict monitoring theory, the adaptation of information processing occurs in a context-sensitive manner in that conflicts signal the need for control recruitment. Starting from the conflict monitoring theory, here the authors discuss the role of affect in the context of conflict-triggered processing adjustments from three different perspectives: (1) the affective value of conflict *per se*, (2) the affective modulation of conflict-triggered processing adjustments, and (3) the modulation of conflict adaptation by reward. Based on the current empirical evidence, the authors stress the importance of disentangling effects of affect and reward on conflict-triggered control adjustments.

Cognitive control refers to the human ability to intentionally carry out a weak response in the face of a dominant but inappropriate response (e.g., Miller and Cohen, 2001). Within the last decade, there have been tremendous research activities within the neuroscientific and psychological disciplines, to learn more about the specific mechanisms underlying such cognitive flexibility. The first main challenge was to identify and understand the processes that inform the cognitive system when and how to implement control without relying on an omniscient homunculus (e.g., Monsell, 1996). In this context, Botvinick et al. (1999, 2001, 2004) in their influential conflict monitoring theory suggested a conflict monitoring module that automatically detects response conflicts in the ongoing processing stream by monitoring the amount of energy over conflicting response nodes.^1^ The elegancy of the assumption is that such a conflict detector does not have to *know* the correct response; it simply registers the *need* for additional control as a consequence of the simultaneous activation of conflicting response tendencies. This conflict information is then sent forward to a control module that biases processing selectivity in accordance with the current task demands. Thus, the detection of a response conflict in trial_N_ triggers the increase of control in trial_N+1_. Behaviorally, this assumption receives support from findings of sequential conflict adjustments in response interference tasks (see Egner, 2007), with the particular finding of smaller response interference effects in trials following conflict than in trials without prior response conflict (e.g., Gratton et al., 1992; Stürmer et al., 2002; Kerns et al., 2004). On a neuronal level, this conflict-control loop is implemented in the anterior cingulate cortex (ACC), that has repeatedly been shown to get activated by response conflicts (Botvinick et al., 1999; Cohen et al., 2000; MacDonald et al., 2000), and the dorsolateral prefrontal cortex (DLPFC) that presumably increases attention to task relevant information, thereby reducing the influence of task irrelevant information (e.g., Kerns et al., 2004; Kerns, 2006).

## The role of affect, reward, and arousal in sequential control adjustments

Recently, Botvinick (2007)—in an attempt to integrate findings showing that the ACC is not only activated by response conflicts but also by monetary loss, social exclusion, pain, and negative performance feedback (e.g., Rainville, 2002; Eisenberger et al., 2003; Nieuwenhuis et al., 2004; Singer et al., 2004)—suggested that the ACC might monitor for any aversive event in general. Aversive signals, from this perspective might then either serve as avoidance learning signal for future action selection (e.g., Holroyd and Coles, 2002; Nieuwenhuis et al., 2004) or as a trigger for processing adjustments (e.g., Kerns et al., 2004; Akcay and Hazeltine, 2007). The idea that the ACC monitors affective and cognitive conflict is not new and has been ascribed to segregate parts of the ACC, namely the ventral ACC for affective conflict and the dorsal ACC for cognitive conflict (Bush et al., 2000). Interestingly, in a recent review, Shackman et al. argued against this segregate model and gathered strong evidence that the anterior midcingulate cortex (aMCC) is conjointly activated by negative affect, pain, and cognitive control (Shackman et al., 2011b). This fits perfectly with the idea that this region of the ACC serves the function of an aversive signal detector in general, as suggested by Botvinick (2007). Importantly, the claim of the ACC as an aversive signal detector has several implications: first, if the ACC registers aversive events, then conflicts *per se* should produce an aversive and thus, most likely affective signal, too. Second, aversive signals should trigger processing adjustments even in the absence of response conflicts if avoidance is not an option, whereas positive signals, on the other hand, should reduce processing adjustments.

So far, there exists evidence for both kinds of modulations. Accordingly, we will start our overview by reviewing the existing literature dealing with the affective value of conflict *per se*. Subsequently, we will present and aim to dissociate affective modulations from reward-based modulations of conflict-triggered processing adjustments, as previous affect and reward studies revealed inconsistent findings (see Table 1 for an overview).

**Table 1:** **Overview of the reviewed studies**.

	**Paradigm**	**Manipulation (affect/reward)**	**Results**
**AFFECTIVE VALUE OF CONFLICTS**			
Brouillet et al. (2011)	Affective priming task	Action-compatible/incompatible trials served as primes prior to the evaluation of target valence	Action-compatible/incompatible trials facilitated the evaluation of positive/negative targets
Dreisbach and Fischer (2012)	Affective priming task	Stroop-trials served as primes prior to the affective evaluation of affective targets	Incongruent/congruent primes facilitated the evaluation of negative/positive targets
Fritz and Dreisbach (in press)	Affective priming task	Stroop-trials served as primes prior to the affective evaluation of neutral targets	Incongruent/congruent primes increased negative/positive judgments of neutral targets
Kool et al. (2010)	Demand selection task	Decks of cards with either high vs. low probability of a task switch served as manipulation of cognitive demand	Anticipated cognitive demand resulted in avoidance behavior (cards from the high demand pile are chosen less frequently)
Schacht et al. (2010)	Simon task	Simon conflict as trigger signal for physiological responses	Simon conflict elicited an EEG conflict signal (N2), but no effects in peripheral measures
Schouppe et al. (2012)	Stroop task	Approach/avoidance responses toward/away from congruent and incongruent Stroop stimuli	Reduced stimulus conflict in the avoidance condition
**AFFECTIVE MODULATION OF CONFLICT ADAPTATION**			
Dreisbach and Fischer (2011)	Fluency task	Number words written in easy and hard to read fonts served as manipulation of aversiveness to trigger processing adjustments	Non-fluent words triggered sequential processing adjustments without any response conflict
Padmala et al. (2011)	Face Stroop	Phasic affect induction (presentation of neutral vs. highly negative pictures with high arousal levels between trials)	Eliminated conflict adaptation for negative pictures (increased interference after conflict trials)
van Steenbergen et al. (2010)	Flanker task	Sustained mood-induction with controlled valence-arousal dimensions	Stronger conflict adaptation after the induction of sad and anxious mood states (irrespective of arousal)
**MODULATION OF CONFLICT ADAPTATION BY PERFORMANCE NON-CONTINGENT REWARD**			
Stürmer et al. (2011)	Simon task	Random presentation of reward and loss cues (Experiment 1)	No effect of random reward and loss cues on conflict adaptation (Experiment 1)
van Steenbergen et al. (2009)	Flanker task	Monetary gain or loss cues as arbitrary reward feedback presented after flanker trials	Gain cues after conflict eliminated conflict adaptation
van Steenbergen et al. (2012)	Flanker task	Monetary gain or loss cues as arbitrary reward feedback presented after flanker trials	Gain cues after conflict eliminated conflict adaptation, conflict related theta oscillations sustained longer after loss
**MODULATION OF CONFLICT ADAPTATION BY PERFORMANCE CONTINGENT REWARD**			
Braem et al. (2012)	Flanker task/Task switching	Performance-dependent reward cues (for fast and correct responses) were presented in 25% of flanker trials (Experiment 1). Switching between Simon and Flanker task (Experiment 2)	Increased conflict adaptation following reward cues (Experiment 1). Reward increased switch costs following conflict trials (Experiment 2)
Stürmer et al. (2011)	Simon task	Performance-dependent reward (for 25% fastest responses) and loss cues (25% slowest responses) (Experiment 2)	Increased conflict adaptation following reward cues (Experiment 2)

## The affective value of conflicts

In two recent studies we could provide evidence in favor of the aversive nature of conflicts. In Dreisbach and Fischer (2012), we adopted the affective priming paradigm (Fazio et al., 1986; Fazio, 2001), in which positive and negative primes ease the affective evaluation of positive and negative targets accordingly. Here, congruent and incongruent Stroop color words served as primes (e.g., RED written in red or in green) and distinct positive and negative words and pictures (e.g., love vs. hate) as targets. Participants' task was to evaluate the affective valence of the targets. In two experiments we found a significant interaction of prime congruency and target valence, showing that—as predicted—positive targets were evaluated faster after congruent Stroop primes whereas negative targets were evaluated faster after incongruent Stroop primes. The results were taken as first empirical evidence for the aversive nature of conflicts. Comparable results have also been reported for action compatible common household objects (Brouillet et al., 2011). In a further study (Fritz and Dreisbach, in press) the authors investigated whether the aforementioned conflict priming effect was actually due to the affective valence inherent in conflict primes or simply due to a match of processing fluency between prime and target (since positive stimuli as well as congruent primes can be processed faster than negative stimuli and incongruent primes). To this end, only neutral targets were presented (words and Chinese characters) and participants' task was to judge spontaneously the affective valence of the (neutral) targets. Results of two experiments showed that neutral targets were more often judged as positive after congruent and more often as negative after incongruent Stroop primes. Results were thus perfectly in line with the first study (Dreisbach and Fischer, 2012) and show that conflict stimuli do not simply share basic processing characteristics with affective stimuli but do indeed convey affective valence.

More indirect evidence for the aversive nature of conflict comes from studies showing that conflict stimuli promote avoidance behavior (Kool et al., 2010; Schouppe et al., 2012). For example, in the Schouppe et al. study, participants were asked to move a manikin on the screen either toward or away from a Stroop color word (depending on its color). The Stroop color words could either be congruent (e.g., BLUE printed in blue), stimulus incongruent (BLUE printed in yellow with blue and yellow affording the same manual response) or response incongruent (BLUE printed in brown, with blue and brown affording different manual responses). The authors found a significant reduction of the stimulus interference effect in the avoidance condition as compared to the approach condition, leading them to the general conclusion that stimulus conflict stimulates avoidance behavior. Interestingly, however, this effect was not found for the response interference condition, which raises the question of how important response execution actually is in order to elicit aversive reactions.

To the best of our knowledge, so far there is only one study that failed to find evidence for the aversive nature of conflict. Schacht et al. (2010) used a Simon conflict task and registered peripheral reactions such as skin conductance response, pupil diameter, and corrugator activation, all of which are known to be sensitive to arousal or affect manipulations. Even though the typical Simon conflict was found in the behavioral data as well as in the N2 component of the EEG, a conflict signal frequently observed in response conflict tasks (Yeung et al., 2004), the peripheral measures were not differentially affected by conflict and non-conflict stimuli.

Taken together, most evidence so far speaks in favor of the aversive nature of conflicts. Furthermore, from the fact that in both conflict priming studies, the primes did not afford an overt response (Dreisbach and Fischer, 2012; Fritz and Dreisbach, in press), together with the observation that only stimulus incongruence (but not response incongruence) enhanced avoidance behavior (Schouppe et al., 2012), it seems that response execution is not that critical a process for conflict to occur as originally assumed in the conflict monitoring model where conflict is computed over the response layer (see Botvinick et al., 2001). However, Botvinick et al. themselves, in their seminal paper, already considered the possibility of conflict at representational levels other than the response level. In fact, they state that conflict in general occurs due to the “simultaneous activation of incompatible representations” (Botvinick et al., 2001, p. 68). In any case, empirical evidence so far suggests that the aversive character of conflicts does not necessarily depend on response execution.

## Affective modulation of sequential conflict adaptation

Another, closely related line of research deals with the effect of affect on conflict-triggered processing adjustments. The first group to directly address this issue was van Steenbergen et al. (2009). They administered an arrow flanker task and arbitrarily presented gain or loss cues after a random subset of trials. It turned out that gain cues following conflict trials eliminated the typical conflict adaptation effect and altered early visual distracter processing (see also van Steenbergen et al., 2012). This was taken as evidence that these cues counteracted the assumed negative valence of conflicts (see Dreisbach and Fischer, 2012; Fritz and Dreisbach, in press) as a consequence of which the conflict adaptation was abolished. These results fit well with a recent study from our laboratories (Dreisbach and Fischer, 2011) where we manipulated the fluency of processing of target words by using either easy or hard to read font. Since there is ample evidence that the experienced ease of processing serves as an affective signal (see Winkielman et al., 2003 for a review), this manipulation made it possible to look into sequential processing adjustments, triggered by aversive (i.e., non-fluent) stimuli even in the absence of any response conflict. Participants had to judge the magnitude of number words that were either written in an easy (fluent) or hard (non-fluent) to read font. Indeed, a significant adaptation effect was found: the fluency effect (non-fluent minus fluent) was smaller after non-fluent than after fluent trials, suggesting that the aversive valence of non-fluent stimuli indeed triggered processing adjustments in terms of increased effort even without any response conflict involved. Finally, in a further study by van Steenbergen et al. (2010), the authors orthogonally manipulated mood and arousal between participants and found a significant interaction of mood and conflict adaptation in the Flanker task: more specially, subjects experiencing calm (positive, low arousal) and happy mood (positive, high arousal) showed a reduced conflict adaptation effect and subjects in a sad (negative, low arousal) and anxious mood (negative, high arousal) showed enhanced conflict adaption, respectively. Arousal, thus, did not have any effects.

So far, results seem to suggest that negative mood (van Steenbergen et al., 2010) or negative stimuli (Dreisbach and Fischer, 2011) promote sequential processing adjustments while positive mood and unconditional reward eliminate conflict adaptation (van Steenbergen et al., 2009, 2010, 2012). However, there is one study that does not fit into the picture. Padmala et al. (2011) presented neutral or highly negative pictures with high arousal levels between picture-word Stroop trials and found reduced conflict adaptation following highly arousing negative pictures. This result obviously stands in sharp contrast to the stronger conflict adaptation effect found by van Steenbergen et al. under sustained negative affect. It remains a question of future research, whether this discrepancy is due to the differential affect manipulations (e.g., Shackman et al., 2006, 2011a) or due to different arousal levels between studies.

## Modulation of conflict adaptation by reward

In contrast to arbitrary reward contingencies of the van Steenbergen studies, reward *conditional* on actual task performance, which on first glance might be closely related to positive affect, appears to have the opposite effects on sequential conflict adaptation. Braem et al. (2012), presented reward cues for fast and correct responses in a flanker task^2^ and hypothesized that conditional reward should *enhance* active connections between stimulus and response, as a consequence of which, conflict adaptation should be amplified. This prediction was derived from the associate learning account of conflict adaptation (Verguts and Notebaert, 2008, 2009; see also Thorndike, 1927). In their theory, and in line with Botvinick's theory, the authors also assume that the ACC detects conflict over the output layer. However, instead of directly sending signals to the DLPFC, the ACC projects to the locus coeruleus which then sends a Hebbian learning signal presumably via increases in noradrenergic activity over the cortex as a consequence of which connections between currently active representations within DLPFC are strengthened. More generally spoken, it is suggested that conflict triggers an autonomic arousal response that strengthens currently active task representations in working memory. By this, the model is able to produce not only the typical sequential conflict adaptation data pattern in terms of reduced response interference on trials following conflict trials, but also the typically observed increased conflict induced switch costs, an effect that the original conflict model would not necessarily predict (Braem et al., 2012, Experiment 2). Importantly, for the present purpose, the associative-learning model predicts that positive signals should serve as a reinforcement signal thereby *strengthening* connections between currently active task representations and thus rather *amplify* conflict-triggered processing adjustments. Results were in line with the authors' hypotheses: conflict adaptation was enhanced following reward cues. Interestingly, within such a reward context, on trials without reward cues *no* conflict adaptation was observed. Unfortunately, no punishment condition was included which makes it hard to decide which of the two data patterns, the *absence* of conflict adaptation following no reward in a reward context or the *presence* of conflict adaptation following reward cues, drives the effect. Support for the authors' interpretation in terms of reinforcement learning^2^ comes from the fact that the effect was further modulated by the sensitivity toward reward (reward responsiveness). Obviously, the results of Braem et al. (2012) thus stand in sharp contrast to the van Steenbergen et al. (2009, 2012) studies reported above. The only difference between both is that the gain cue in van Steenbergen's studies (2009, 2012) was entirely random and not contingent on behavior whereas in the Braem study, participants were informed that on a predetermined number of trials, reward could be earned for fast and correct responding. This might have rendered the gain cue in the van Steenbergen study a simple positive affect cue that counteracted the aversive nature of the preceding conflict, whereas the reward cue in Braem's study informed about the successful completion of the preceding response. Fortunately, there is one study that directly investigated the effects of random versus performance contingent gains and losses on conflict adaptation. Stürmer et al. (2011) presented gains and losses randomly and non-contingent on the respective performance in one experiment and compared the effects to a second experiment, where only the 25% fastest and the 25% slowest responses were rewarded and punished, respectively. It turned out that random gains and losses had *no* effect on conflict adaptation (Experiment 1). In contrast, gains contingent on fast and correct responses enhanced conflict adaptation effects (Experiment 2). The results of the Stürmer et al.'s study (2011) are thus in line with Braem et al. (2012) and support the assumption that reinforcement that is contingent on actual task performance *strengthens* active connections between task representations and the response. Random gains irrespective of task performance, on the other side, seem to have either *no* effect (Stürmer et al., 2011) or even *eliminate* conflict adaptation (van Steenbergen et al., 2009). This assumption might also explain other seemingly contradictory results from two studies in a related field using the AX continuous performance task, a paradigm well-suited to study processes of goal maintenance. Whereas positive affective pictures (non-contingent on performance) *reduced* goal maintenance (Dreisbach, 2006), in the same paradigm, reward *improved* goal maintenance (Locke and Braver, 2008).

Given the findings of eliminated conflict adaptation under positive affect and unconditional reward (van Steenbergen et al., 2009, 2010, 2012) one might therefore speculate that random gains also produce a positive affective reaction that, however, is different from the affective reaction due to successful task performance. One possible reason could be that performance contingent reward increases the intrinsic reinforcement signal in response to correct responses (Satterthwaite et al., 2012). Such intrinsic reinforcement signals are elicited within the ventral striatum, a key region of dopamine function, are stronger for correct than incorrect responses and are further modulated by task difficulty (Satterthwaite et al., 2012). From this perspective, successful conflict resolution itself might trigger an intrinsic reinforcement signal (cf. Braem et al., 2012) which might further be enhanced by external performance contingent reward. Non-contingent random reward, on the other side, might actually counteract the intrinsic reward signal, as it presumably conveys the information that task performance is not a value by itself.

## Conclusion

In this short review, we first presented evidence from different studies showing that conflict signals are registered as aversive. Second, while most studies seem to suggest that positive affect as subjective experience *reduces* conflict-triggered processing adjustments, reward as motivational manipulation, on the other hand, appears to *strengthen* conflict-triggered processing adjustments. Based on the present literature we suggest that unconditional reward reduces the intrinsic reward signal whereas positive mood reduces the negative experience of the conflict signal—both resulting in reduced conflict adaptation. Reward contingent on task performance, by contrast, may serve as reinforcement signal, enhancing bindings between currently active task representations and response—thereby increasing conflict adaptation. We thus close this review by emphasizing the importance to empirically and theoretically disentangle effects of affect and reward on processes of cognitive control in general and on conflict adaption in particular (see also Chiew and Braver, 2011).

### Conflict of interest statement

The authors declare that the research was conducted in the absence of any commercial or financial relationships that could be construed as a potential conflict of interest.

## Footnotes

^1^In this review we will focus on conflict-triggered control adjustments, but acknowledge that other signals, e.g., errors (Holroyd and Coles, 2002), may also induce processing adaptations.

^2^In fact, the authors ran a second experiment using the task switching paradigm. For task switching, the associative learning account (ALA) makes the opposite prediction, namely that reward increases switch cost following conflict trials because on task switches following a reward signal, the formerly relevant task and its corresponding connections become strengthened as a result of the reinforcement signal. The results were as predicted by ALA.
